# The Mother

**Published:** 1878-07

**Authors:** 


**The Mother.**—She came leaning on the arm of her daughter, and wrapped in a thick cashmere shawl, which alone indicated the extreme delicacy of a constitution that could not endure exposure to a breeze so gentle as that which pervaded the apartment. One needed to bestow but a moment's glance on the mother to see whence the mountain girl inherited the spiritual expression which at times imparted such holy sweetness to her face. Nothing could exeeed the elevated, the almost unearthly sanctity which marked the countenance, the manner, and even the voice of the slender, shadow-like woman, the marble pallor of whose face seemed enhanced by the brilliancy of her dark, lustrous eyes, and whose black, wavy hair drooped over her sunken cheek as if it were a mourning badge, a token of the decay of her early bloom. There was no undue claim to sympathy, however; no affectation of weakness in the gentle, hostess-like manner of the invalid, who, although she spoke English but imperfectly, made a successful use of her knowledge of the language in welcoming Meredith under her roof, accompanying her broken words with a kindness of tone and earnestness of gesture which left little for the tongue to express.

Mother—O word of undying beauty! Thine echoes sound along the walls of time until they crumble at the breath of the Eternal. In all the world there is not a habitable spot where the music of that holiest word is not sounded. Ay, by the golden flower of the river, by the crystal margin of the forest tree, in the hut built of bamboo-cane, in the mud and thatched cottage, by the peaks of the kissing mountains, in the wide-spread valley, on the blue ocean, in the changeless desert, where the angel came down to give the parched lips the sweet water of the wilderness; under the white tent of the Arab, and in the dark-covered wigwam of the Indian hunter—wherever the pulses of the human heart beat quick and warm, or float feebly along the current of failing life, is that sweet word spoken like a universal prayer.

